# Factors influencing nurses participation in the health policy-making process: a systematic review

**DOI:** 10.1186/s12912-021-00648-6

**Published:** 2021-07-12

**Authors:** Alireza Hajizadeh, Vahid Zamanzadeh, Edris Kakemam, Rona Bahreini, Rahim Khodayari-Zarnaq

**Affiliations:** 1 Department of Health Economics and Management, School of Public Health, Tehran University of Medical Sciences, Tehran, Iran; 2 Department of Medical Surgical Nursing, School of Nursing and Midwifery, Tabriz University of Medical Sciences, Tabriz, Iran; 3 Department of Health Services Management, School of Health, Qazvin University of Medical Sciences, Qazvin, Iran; 4 Student Research Committee, School of Management and Medical Informatics, Tabriz University of Medical Sciences, Tabriz, Iran; 5 Department of Health Policy and Management, School of Management and Medical Informatics, Tabriz University of Medical Sciences, Tabriz, Iran; 6 Tabriz Health Services Management Research Center, Health Management and Safety Promotion Research Institute, Tabriz University of Medical Sciences, Tabriz, Iran

**Keywords:** Nurse, Participation, Health policy, Systematic review

## Abstract

**Background:**

Nurses as the majority of the health care workforce help in the health systems strengthening. Nurses’ involvement in health policy making is clear; however, still few are involved in policy-making processes, even in the clinical context. The aim of the present systematic review was to identify factors influencing nurses participation in the health policy-making process.

**Methods:**

The present systematic review was designed on studies conducted between 2000 and 2019. Four online databases including PubMed, EMBASE, SCOPUS and Science Direct were searched using comprehensive terms. Study selection, quality assessment, data extraction, and data analysis were independently done by two reviewers. Inclusion criteria included published studies in English language and between 2000 to 2019, participants such as nurses and the healthcare managers, mentioned influential factors, types of participants were included nurses and the healthcare managers, study designs and methods clearly defined. The methodological quality of included article was appraised using the checklists of CASP and MMAT. Finally the data were analyzed using content analysis.

**Results:**

After quality assessment, 11 studies, according to inclusion criteria, were retrieved. Nine studies had a good, 2 a medium, and non-articles was poor methodological quality. Three main themes include nursing-related factors (4 sub- themes), management and organizational factors (8 sub-themes) and creating a positive work environment (3 sub-themes) identified as affecting factors on nurses participation in health policy.

**Conclusion:**

Nurses can utilize this finding to develop empowering programs to play efficient roles and increase their participation in health policy making. Also, the extracted factors in this review can place nurses in suitable position and make them potential agents in changing the ways of policy-making. Further studies are required to survey the relation between these factors and nursing participation in health policy making.

## Background

In rapidly changing and developing health systems, nurses comprise the major group of health care personnel who are mainly responsible for providing people with qualitative care [1]. The political, environmental, technological and financial pressures in healthcare systems affect all practical settings. These changes can make opportunities for all personnel, especially nursing staff to enhance their position and role in healthcare policies and management [2–4].

According to the definition by World Health Organization (WHO), health policies refers to decisions, plans, and actions undertaken to achieve specific health care goals within a society [5]. Also WHO emphasized that health policy and practice require actions form multi-sectors, and decisions that are made in these sectors must be responsive and sensitive to the concern of health [6]. The ultimate goal of health policies is to promote public welfare. It consists of three stages: formulation, policy implementation and policy reformation [7, 8]. Health policy is a tool which nurses must utilize to improve the safety and quality of healthcare [9].

When elaborating on health policies, there must be a motivation for nurses to participate in health policy-making processes. For example, nurses can have influence through their experiences on policies, laws, and regulations that govern the healthcare system [10, 11]. Nursing Staff are encouraged to participate in health policy for three reasons. First, nurses closely deal with patients and their families in a variety of settings; therefore, their comments can be considered as valuable sources for policy development. Second, different health policies have direct effects on nurses. Thus, policies should ensure a supportive work setting. Third, nurses play key role in professional development and can highly contributed to the formation of appropriate and efficient health policies [12, 13].

The International Council of Nurses (ICN) strongly emphasizes and supports those efforts in improving nurses’ readiness in developing policies [12]. Previously, the ICN has tried to address topics such how health policy is made, different approaches to making policy and how nurses and professional organization can influence policy [14]. Also, in issues of nursing such as health policy, the global Nursing Now campaign is working with the ICN, and the WHO, to create and strengthen strategic nursing leadership, as modelled by the International Council of Nurses’ Global Nursing Leadership Institute [15]. Different factors affect nurses’ ability to be active in health policy development including gaining experience in policy development process, gaining knowledge on health systems, policy research and developing leadership skills [1, 16]. In recent decades, despite the fact that nurses have become increasingly knowledgeable, skilled, and well-educated, they have had limited involvement in policy making processes and political decisions,affecting the delivery of health services [13, 15]. Also, nurses’ ongoing work in the COVID-19 pandemic is making nursing history and there are significant opportunities to learn from this pandemic, to find better ways of doing things in practice, and contributing to policy-making through evidence-based research and empowerment strategies [17].

A study conducted in Thailand showed that most nurses are involved only in the implementation of health policies while it is essential issue that they must gain perception over the issue and actively participate in it [1]. Abu-Al-Rub & Foudeh conducted a study to evaluate the level of involvement of Jordanian nurses in the development of health policy and perceived benefits, barriers, and impacts on health outcomes of involvement in health policy process. Their results indicated that the low level of Jordanian nurses’ involvement in health policy can be attributed to the fact that most participants, beside their roles in workplace, had family roles making them to allocate little time for health policies activities. Lack of mentoring by nursing leaders could also negatively affect their involvement in health policies development [5]. In study by Shariff & Potgiete it was demonstrated that facilitators of health policy development comprise having knowledge and skills, enhancing the image of nursing and enabling structures and processes. Also, barriers to the participation of nursing leaders include the lack of involvement, insufficient knowledge and skills, negative image about nursing, lack of dynamic structures and insufficient resources [18].

Nurses’ participation in national policy making processes is significantly important in Low- and Middle-Income Countries (LMICs) where nurses comprise larger proportion of the health sector workforce. Thus, there is a need to enhance their ability in understanding, generating, and utilizing research knowledge that is beneficial for making changes in policy [16, 19, 20]. Several studies have emphasized that contemporary nurses influence health policies. The purpose of this systematic review was to designing a framework for nursing participation in health policy making. So, the research question were developed by authors was as follows:

- What factors are associated with success of nurses for participation in health policy making?

## Methods

### Study desigen

The present systematic review was designed and conducted in a time span of 5th to 10th of July, 2019. We searched using a modified form [21] of the SPIDER tool developed by Cook and colleagues (Table 1). The SPIDER tool used to identify relevant qualitative and mixed-method studies. Also, the addition of the “design” and “research type” categories to the SPIDER tool was intended to further increase the ability of this tool to identify qualitative articles [22].

**Table 1:** Breakdown of the research question

SPIDER heading	Search topics
S – sample	Nurses
PI – phenomenon of interest	Participation in health policy making
D & R – design and research type	qualitative research

### Search strategy and data sources

Data were gathered by searching the four online databases including PubMed, EMBASE, SCOPUS and Science Direct. Relevant article were identified by two reviewers (A.H. and R.B.) independently, and search algorithm varied according to the specifications of each database. To identify the additional relevant articles being lost in the database search, we checked the references of the selected publications (reference by reference). A summary of search strategy based on keywords are outlined in Table 2.

**Table 2:** Search strategy

Database	Search terms	Number of articles
PubMed	(participation OR involvement OR contribution OR involution OR engagement OR activation) AND (nurse*) AND (“health policy” OR “health policies” OR “policy making”)	377
EMBASE	(participation OR involvement OR contribution OR involution OR engagement OR activation) AND (nurse*) AND (“health policy” OR “health policies” OR “policy making”)	249
SCOPUS	(participation OR involvement OR contribution OR involution OR engagement OR activation) AND (nurse*) AND (“health policy” OR “health policies” OR “policy making”)	565
Science Direct	(participation OR involvement OR contribution OR involution OR engagement OR activation) AND (nurse*) AND (“health policy” OR “health policies” OR “policy making”)	19
Total articles		1210
Total articles obtained from reference by reference		1
Total abstract and titles reviewed (duplicates removed)		585
Total articles papers reviewed		57
Selection of studies		11

### Inclusion and exclusion criteria

In this review, studies were included if they: (1) were published in English language and (2) between January 2000 to August; 2019 (3) were focus on the involvement of nursing in the health policy making (4) types of participants and experts were included nurses and the healthcare managers at all levels of management (men and women) worked at healthcare institutions or organizations (5) design and method clearly defined and (6) reported to factors effect nursing participation in health policy making. On the publication year criteria, due to the expiration of studies published before year 2000, time span of 2000 to 2019 was selected for the present review.

exclusion criteria included: (1) outcome reported were ambiguous and was ineligible for the data synthesis (2) evaluation results fall within the scope of low quality research after using the checklist quality assessment tool (3) editorials (4) letters to the editor (5) protocol (6) commentaries and (7) conference abstracts. Moreover, if an articles did not unavailable full-text download link the corresponding authors were contacted via e-mail to ask for full-texts and was excluded in case of non-responding.

### Study selection and quality assessment

After preliminary selection of articles by the first reviewer (A.H.) and their verification by the last reviewer (R.B.) the duplicated studies were excluded. Two reviewers (A.H. and R.B.) independently screened the titles, abstracts and full-text of the articles.

The Critical Appraisal Skills Program (CASP) checklist was applied for qualitative assessment of the studies [23] that were evaluated by two reviewers (A.H. and R.B.). The tool consisted of 10 questions on methodology and components of the article. The Mixed Methods Appraisal Tool (MMAT) to appraise mixed method studies was also implemented. MMAT is a critical appraisal tool designed for the appraisal stage of systematic mixed studies reviews, i.e., reviews that include qualitative, quantitative and mixed methods studies [24]. In case of disagreement, third-party opinions were asked to reach consensus. To scoring the quality of the final studies, they were divided into three categories: poor (0–3), medium (4–7) and good quality (8–10). Finally, studies with poor quality were excluded.

### Data extraction

Data extraction table included: author, publication year, country, design of study, method of data collection, quality assessment and factors affecting nursing participate in health policy making. In this stage two authors (A.H. and R.B.) independently extracted data from the included articles. In case of disagreement between two reviewers (A.H. and R.B.), a third reviewer (R.KH.Z) was involved to make a final decision.

### Data synthesis

Content analysis carried out for data analysis. Based on the concense of authors, we chosed content analysis method to quantify and analyze the presence, meanings and relationships of such certain words, themes, or concepts. Also, content analysis is used to make replicable and valid inference that allows the researcher to identify specific characteristics of messages [25]. First of all, the content of each extracted text was broken into meaningful units as codes. Then, the cods were categorized according to their similarities and differences. After interpreting the content in each category, the main themes were identified. A total of 3 themes and 15 sub-themes were obtained. Two researchers (A.H. and R.B.) conducted analysis independently to establish the credibility (peer check).

## Results

### Results of the search strategy

**Fig. 1:**
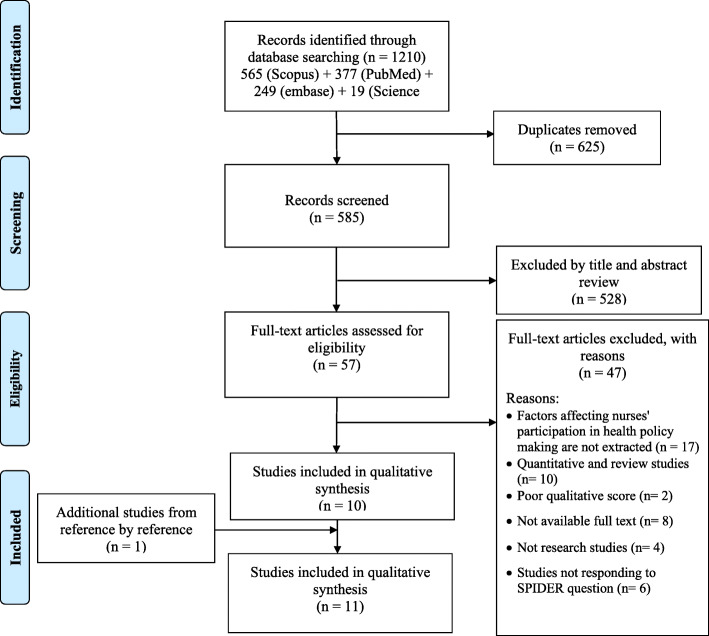
PRISMA Flow Diagram of literature search process

The search process yielded for papers has been summarized in Fig. 1. Of 1210 potentially relevant articles reviewed, 625 records were duplicated. After excluding the irrelevant articles by title and abstract review (528) and applying exclusion criteria (47), 10 studies remained. One additional study was identified through reference by reference and included in qualitative analysis. Finally, 11 articles were included in this systematic review.

### Description of included studies

Eleven studies were included from 18 countries: Kenya (*n* = 4), USA (*n* = 2), Iran (*n* = 2), South Africa (*n* = 2), Uganda (*n* = 2), Canada (*n* = 1), UK (*n* = 1), Thailand (*n* = 1), Tanzania (*n* = 1), Jamaica (*n* = 1) and Barbados (*n* = 1) and they were published from 2001 to 2017 in different journals. Summary of the characteristic of included studies are depicted in Table 3. Content analysis was conducted for each three main themes and was categorized into 15 sub-themes. The results of content analysis are presented in Table 4. This Table also shows the frequency of extracted factors.

**Table 3:** Summary characteristic of included studies

Study (year)	Country	Design of study	Participants (n)	Method of data collection	Quality assessment
Gebbie et al., 2000 [20]	USA	Qualitative study	Nurses (27)	Semi-structured interviews	Good
Deschaine and Schaffer, 2003 [21]	USA	Qualitative study	Assistant director, director and administrator (8)	Semi-structured interviews	Medium
Kunaviktikul et al., 2010 [1]	Thailand	Mixed method	Nurses (2121) and Nurse leaders (26)	Questionnaire and interview	Good
Richter et al., 2013 [13]	Canada, Jamaica, Barbados, Kenya, Uganda and South Africa	Mixed method	Nurses (51)	Interview	Good
Juma et al., 2014 [12]	Kenya	Mixed method	Non nursing decision-makers, national level nurse leaders, frontline nurses and frontline managers (32)	Open-ended interview	Good
Ditlopo et al., 2014 [22]	South Africa	Qualitative study	Informants (28) and nurses (73)	Semi-structured interviews	Good
Aarabi et al., 2015 [26]	Iran	Qualitative study	experienced nurses (17)	deep semi-structured face to face interviews	Good
Shariff, 2014 [15]	Kenya, Uganda and Tanzania	Mixed method	Nurse leaders (78)	Questionnaire and experts panel	Good
Cheraghi et al., 2015 [24]	Iran	Qualitative study	Nurse leaders from all levels of management or administration (22)	Semi structured face to face in depth interviews	Good
Shariff, 2015 [27]	Kenya	Mixed method	Nurses (78)	Questionnaires and Experts panel	Good
O’connor, 2017 [28]	UK	Mixed method	All National Health Service (NHS) regional boards and higher education institutes as well as numerous related voluntary and government agencies associated with providing nursing and health services in Scotland	Virtual focus group posthumously using the hashtag #CNOScot	Medium

**Table 4:** Themes and sub-themes of factors affecting nurses participate in health policy making

Main themes	Sub-themes	Factors
Nursing related factors	Nurses’ viewpoints on policy making	• Lack of priority of health policy for nurses (1) • Not knowing the process of health policy as part of the tasks (1) • The existence of psychological issues in active participation (2) • Disapproval of nurses’ involvement in policymaking by others (2)
	Lack of proper reaction by nurses	• Fear of facing different perspectives (1) • Fear of confrontation with administration (1) • Feeling powerless (2) • Fear of facing new challenges (2)
	Gaining experience and skills	• Lack of skills to engage in process of health polices (5) • Building experience in the nursing (2) • Limited skills in public relations (3) • Improvement of their political skills (4) • Lack of research skills (4)
	Education and research system	• Lack of knowledge and education about the policy-making process (4) • Lack of understanding of a complex political process (4) • Lack of public understanding (3) • Lack of access to research resources (3) • Personal interest in political knowledge and information (3) • Integrating Political education in the design nursing curriculum (3) • Undertaking research in health policy (3) • General deficiencies in nursing education (2) • Inability of nurses to bring forward research evidence to inform policy formulation (1) • Clarity in research directions (1) • Building of supportive research environments (2) • Strengthening nurses’ research capacity (2) • Promotion of evidence based decision making in nursing practice (1) • Providing International training opportunities for research and policy formulation (1)
Management and organizational factors	Creating communication networks	• Lack of communication from the top down (4) • Lack of communication networks and bonding (2) • Lack of sense of teamwork and collegiality (3) • Lack of involvement with nursing organizations (1) • Lack of national nursing association (1) • Lack of professional interest groups (1) • Use of technology and informatics (2) • Lack of collective action amongst different nursing stakeholders (2) • Existence of unity (2)
	Gaining and sharing knowledge and information	• Lack of access to information (2) • Lack of university nursing academics (2) • Lack of college nursing educators (1) • Lack of information sharing (2) • Lack of sharing of policies (1) • Getting new ideas from nursing literature (1) • Utilizing evidence based information (2)
	Providing specialized and motivated human resources	• Lack of access to key individuals (2) • Increasing number of PhD nurses (1) • Having an organizational commitment (1) • Nurses motivation (1) • Shortage of nursing (2)
	Providing non-human resource	• Lack of time (5) • Lack of money and other resources (8)
	Establish effective leadership styles	• Combination of proactive leadership (1) • Leadership development among nurses (1)
	Establishment of incentive organizational structure	• Follow Health policy of top-down approach (2) • Lack of enabling structures (5) • Sufficient authority (1) • The existence of a hierarchical system (2) • Bottom-up approach (2) • Clinical governance (1) • Determining where the power lies in organizations (2)
	Membership in advisory and policy making committees	• Lack of nurses’ membership in the Policy development committee (1) • Membership in professional and or advocacy organizations (1)
	Health policy outcomes and impact	• Correct role playing of policy making by nurses (1) • Lack of health policy capacity (1) • Dispersion in making policies (1) • Different attitudes of health policy makers (2) • Failure to make a difference by political activities (2)
Work environment	Environmental elements	• Existence of mental health issues among nurses (2) • The negative image of nursing by others professions (3) • Creation of an enabling environment (2) • Creating more opportunities for participation of nurses (2)
	External support	• Lack of support from political sector (2) • Lack of support from government officials (1) • Lack of support from professional organizations (1) • Strong support from the national nursing association (1) • Non-academic view of some physicians on the field of nursing (2)
	Establishing fair and right work rules	• Heavy workload (2) • Career development of nurses (1) • Sex issues (2) • Restrictions of Nursing recruitment (1)

### Study quality

All 11 studies were classified to have poor, medium or good in quality. After quality assessment, nine studies (81.82%) had good quality, two (18.18%) had medium quality. Also the quality level of non-articles was poor.

## Discussion

In this systematic review we have tried to identify factors affecting nurses participation in health policy making. The effective factors are developing and changing over time. The total of 11 included studies published from 2000 to 2019 indicates these common factors. Most of the studies were homogenous in terms of purpose and data collection methods. It is not precisely clear why nurses do not become involved in health policy making, according to our there is consensus over several factors. These factors include sources limitations, insufficient time, political knowledge, heavy workloads, and gender issues, negative images about nurses, management supports, and fear to encounter with others’ beliefs that negatively affect nurses’ involvement in policy making making.

The findings of present review revealed that insufficient knowledge on the health policy making is one of the important reasons of nurses’ non-involvement in health policy making making. The findings also indicated that insufficient knowledge and skills on evaluation of policy and insufficient knowledge on the health policy formulation guidelines are barriers to the nurses’ participation in health policy making [13, 18].

Lack of resources was most frequently mentioned factor in studies. Lack of available resources was identified as a factor for the participation of nursing leaders in health policy making [5, 18]. According to our findings, gaining external support was mentioned as one of the sub-themes for creating a positive work environment. Lack of support on behalf of different sectors such as the political sector, government officials, or professional organizations were obstacles in low involvement of nurses in policy making [1].

Based on the results of our review, education and research system, as a nursing-related sub-theme could affect nurses’ participation. Researches in, PhD curriculum in nursing and training were regarded as the facilitators of nurses’ involvement in policy making processes in LMICs [26, 27]. Parallel to our study, the study of O’connor [28] showed that education and research are necessary to develop nursing workforces’ participation. Also, the finding of a study supported that active learning increases retention and utilization by faculty at schools of nursing in the area of health policy among nurses [29]. Preparing nursing students to particpate in health policy is critical to ensuring the nursing workforce can effect the plans, structure and financing of health care. The findings of study in USA nursing students reported high levels of involvement in some health policy activities and provide insight into how nursing educators and administrators can capitalize on students’ experiences and interests to better prepare nurses in health policy [30].

Most of the factors that affect nurses’ participation are related to management and organizational factors, to which the included studies point to their importance [13, 16]. Supportive organizational structure is a prerequisite for the establishment of policy making activities.

Establishing communication networks was identified as one of the factors affecting nurses’ participation in the policy making processes [16]. This network largely focuses on interacting with internal organizational members and interested external publics. Leadership styles are categorized as one of the sub-themes of management and organizational factors [1]. This factor is dependent on the organizational structure according to the framework presented. Based on the findings, health policy outcomes and impacts are considered as a supportive system for establishing organizational structure. Many studies have mentioned the importance of this factor in increasing the nurses’ participation [31].

This study provide valuable results for decision makers and policymakers to engage nurses in their affaires. Understandings factors that affect nurses’ participation in health policy making can offer insight about strategies to strengthen nurses’ role in health policy.

Nurses’ inadequate participation in policy-making processes is going to continue in the future and in many countries. Nurses need to understand the importance of empowerment and participation in the policy-making process. This review identified key factors that will help nurses to have an active and productive participation. Also, it leads to long-term benefits in workplaces. Despite progressions in in nurses’ skills and knowledge, there is a gap in the active involvement of nurses in health policy-making processes. Identifying the barriers and facilitators can help nurses to play an effective role in formulation, implementation and reformation of health policies. These include creating a context for nurses to communicate with policymakers, reducing the burden of their workload and using appropriate leadership approaches which all can help nurses in this regard. Also, the extracted factors can be applied in the development of educational programs on improving nurses’ knowledge and skills.

Based on our findings, the researchers recommend that nursing managers and professors should focus on the empowerment and reinforcement of nurses in all fields such as clinical, educational, and skill and communication. Future studies need to examine the relation between effective factors and nurses’ participation in policy making which can identify the needs and the fields of improvement. In order to enhance nurses’ participation in health policy-making processes, leadership and political competence are suggested. Also the future studies should investigate the impact of the factors extracted from the studies on nursing roles.

### Limitations

Lack of fluency in other languages to use the results of non-English language studies was one of the most important limitations. Another limitation was inaccessibility to some other databases such as CINAH and Web of Science. Also, we had no access to unpublished dissertations and full-text of some articles.

## Conclusion

In the health care providing systems, due to the fact that different health policies can directly affect the role of nurses, thus, they need to have more participation in health policy making. Nurses’ failure to involve in policy making has led to be a concern of WHO and ICN. Increasing the capability of nurses to participate in policy-making activities is an important aspect of constant promotion of health services. The level nurses’ engagement in policy-making processes can be enhanced by having more focus on the improving of health policy education and competency as facilitator, as well as by overcoming the barriers such insufficient resources and skills. In this review we identified those affecting factors that support nurses’ participation in policy making by elaborating on the ways of increasing their policy-making activities. Findings of this study and developed framework can be efficient in empowering nurses to create active role and better’s future through the development of policy-making activities. Also, the extracted factors in this review, and even more, can place nurses in suitable position and make them potential agents in changing the ways of policy-making.

## Data Availability

Datasets are available through the corresponding author upon reasonable request.

## Abbreviations

WHO: World Health Organization
ICN: International Council of Nurses
LMICs: Low- and Middle-Income Countries
SPIDER: Sample, phenomenon of interest, design, evaluation, research type
CASP: Critical Appraisal Skills Program
MMAT: Mixed Methods Appraisal Tool
